# Fungal Endophytes as Efficient Sources of Plant-Derived Bioactive Compounds and Their Prospective Applications in Natural Product Drug Discovery: Insights, Avenues, and Challenges

**DOI:** 10.3390/microorganisms9010197

**Published:** 2021-01-19

**Authors:** Archana Singh, Dheeraj K. Singh, Ravindra N. Kharwar, James F. White, Surendra K. Gond

**Affiliations:** 1 Department of Botany, MMV, Banaras Hindu University, Varanasi 221005, India; singh.archi90@gmail.com; 2 Department of Botany, Institute of Science, Banaras Hindu University, Varanasi 221005, India; 3 Department of Botany, Harish Chandra Post Graduate College, Varanasi 221001, India; 4 Department of Plant Biology, Rutgers University, New Brunswick, NJ 08901, USA

**Keywords:** endophytic fungi, medicinal plants, plant-derived, host-derived, bioactive, natural products, secondary metabolites, drug discovery

## Abstract

Fungal endophytes are well-established sources of biologically active natural compounds with many producing pharmacologically valuable specific plant-derived products. This review details typical plant-derived medicinal compounds of several classes, including alkaloids, coumarins, flavonoids, glycosides, lignans, phenylpropanoids, quinones, saponins, terpenoids, and xanthones that are produced by endophytic fungi. This review covers the studies carried out since the first report of taxol biosynthesis by endophytic *Taxomyces andreanae* in 1993 up to mid-2020. The article also highlights the prospects of endophyte-dependent biosynthesis of such plant-derived pharmacologically active compounds and the bottlenecks in the commercialization of this novel approach in the area of drug discovery. After recent updates in the field of ‘omics’ and ‘one strain many compounds’ (OSMAC) approach, fungal endophytes have emerged as strong unconventional source of such prized products.

## 1. Introduction

Several recent reports suggest that natural products may play a substantial role in the drug discovery and development process as a source of diverse and novel templates for future drugs [1,2,3,4]. With the rapidly evolving recognition that significant numbers of natural products are either produced by microbes or a result of microbial interactions with their hosts, the area of endophyte research for natural products is positioned to take the drug discovery and development process to the next level [5,6]. In the backdrop of the past 25 years of studies, endophytes may be defined as a polyphyletic group of unique microorganisms residing in healthy living internal tissues of the plants with covert and/or overt positive effects on their hosts. They establish a variety of intricate biological intra- and inter-relationships among them and with their hosts, respectively. Endophytes are able to produce a multitude of secondary metabolites with diverse biological activities [7,8,9]. However, merely 0.75–1.50% of known plant species has been explored for their endophytes yet. So, the opportunity to find new potential bioactive metabolites from cryptic endophytic microorganisms of nearly 374,000–400,000 plant species congruently occupying millions of biological niches is considered high [5,10]. This opportunity has increased further with the innovative discovery of biosynthesis of *Taxus* derived anticancer compound ‘taxol’ from its endophytic fungus *T. andreanae* in 1993 by Stierle et al. [11]. This discovery leads to renewed attention in endophytic fungi for isolating plant-derived medicinal compounds [12,13,14]. Later, a series of works revealed that a reasonable number of plant-derived compounds are synthesized by endophytes rather than hosts [9,15]. However, there are unsettled and contradictory reports regarding the phylogenetic origin of genes related to the biosynthesis pathway of such plant-derived compounds in host plants and their microbial endophytes [16]. The above facts prompted us to use the word “plant/host-derived” rather than “plant/host-origin” for such compounds. Nevertheless, it is now an established fact that endophytes can co-/produce, induce, and/or modify a plethora of “specific plant-derived” metabolites in-/outside of host plants [12,14,17]. Such discoveries opened the new horizons for the up-scaled production of plant-derived medicinal compounds from endophytes. The recent increase in demand for natural products and difficulties in accessing them from plants make endophytes interesting targets for the assessment and isolation of typical host-derived compounds [18,19]. Since medicinal plants are an inherent source of many therapeutic compounds, it is vital to explore their endophytes to isolate such compounds. The current review aims to provide an up-to-date overview on the globally isolated specific plant-derived bioactive compounds synthesized by fungal endophytes from the period 1993 to mid-2020. It will also focus on applications and modes of actions of such compounds. This review will also provide insights about different challenges in employing endophytes as an alternative source for the synthesis of plant-derived bioactive compounds and their application in drug discovery. Its outcome would certainly lead to strategize the use of endophytes as an efficient novel source for plant-derived metabolites. 

## 2. Plant-Derived Bioactive Natural Products from Fungal Endophytes

A wide array of secondary metabolites in fungi is biosynthesized from very few key precursor compounds by slight variations in basic biosynthetic pathways and can be classified into nonribosomal peptides, polyketides, terpenes, and alkaloids. Nonribosomal peptides are biosynthesized by multimodular nonribosomal peptide synthetases (NRPS) enzymes using both proteinogenic and nonproteinogenic amino acids. Polyketides are biosynthesized by polyketide synthase (PKS) enzymes from acetyl-CoA and malonyl-CoA units. Terpenes consisting of isoprene subunits are biosynthesized from the mevalonate pathway catalyzed by terpene cyclase enzymes. Alkaloids are nitrogen-containing organic compounds biosynthesized as complex mixtures through the shikimic acid and the mevalonate pathways, as they are usually derived from aromatic amino acids and dimethlyallyl pyrophosphate [20]. Other classes of fungal secondary metabolites are linked with the above four groups of compounds. For ease and better understanding, we have classified different fungal secondary metabolites as alkaloids, coumarins, flavonoids, lignans, saponins, terpenes, quinones, and xanthones, and miscellaneous compounds. Coumarins are a class of lactones consisting of a benzene ring fused to a α-pyrone ring and are mainly biosynthesized by the shikimic acid pathway from cinnamic acid. Flavonoids are synthesized by the phenylpropanoid pathway from phenylalanine using enzymes phenylalanine ammonia lyase (PAL), chalcone synthase, chalcone isomerase, and flavonol reductase [21]. Lignans are low molecular weight polyphenols biosynthesized by enzymes pinoresinol-lariciresinol reductase (PLR), PAL, cinnamoyl-CoA reductase (CCR), and cinnamyl-alcohol dehydrogenase (CAD) [22]. Saponins are glycosides containing a non-sugar triterpene or steroid aglycone (sapogenin) attached to the sugar moiety. Saponins are derived from intermediates of the phytosterol pathway using enzymes oxidosqualene cyclases (OSCs), cytochromes P450 (P450s), and UDP-glycosyltransferases (UGTs) [23]. Quinones are biosynthesized through several pathways; for example, isoprenoid quinones are synthesized by the shikimate pathway using chorismite-derived compounds as precursors, terrequinone by NRPS from L-tryptophan, dopaquinone by tyrosinase from tyrosine, and benzoquinone by catechol oxidase/PKS from catechol [24]. Xanthones comprise an important class of oxygenated heterocyclics biosynthesized through the polyacetate/polymalonate pathway by the internal cyclization of a single folded polyketide chain [25].

### 2.1. Plant-Derived Alkaloids from Fungal Endophytes

After a systematic literature survey, we enlisted 19 plant-derived medicinal alkaloids that have been produced by different endophytic fungi (Table 1), and some important alkaloids are described below.

#### 2.1.1. Aconitine

Aconitine, a diterpenoid alkaloid found in *Aconitum* spp., is a voltage-gated sodium channel activator that effectively opens the Na^+^ channels causing the prolonged presynaptic depolarization of muscles and neurons. In Chinese folk medicine, aconitine is used for pain relief caused by trigeminal and intercostal neuralgia, rheumatism, migraine, and general debilitation. Aconitine is a strong cardiotoxic and neurotoxic agent, and its side effects may cause bradycardia, hypotension, ventricular dysrhythmia, and inhibition of the release of neurotransmitters [82]. Aconitine is also synthesized by endophytic fungus *Cladosporium cladosporioides* from *Aconitum leucostomum* [26].

#### 2.1.2. Berberine

Berberine, an isoquinoline alkaloid found in *Berberis* spp. and some other plants (Table 1), is widely used in the treatment of hyperglycemia, hyperlipidemia, gastrointestinal, cardiovascular, renal, and neural disorders. The antidiabetic efficacy of berberine is comparable to that of the popular drug metformin. Its hypoglycemic effect is exerted via inhibition of mitochondrial function, stimulation of glycolysis, activation of AMP-activated protein kinase (AMPK)/AMPK pathway, and increasing insulin sensitivity. Moreover, berberine has additional advantageous effects on diabetic cardiovascular complications due to its antihypercholesterolemic, anti-arrhythmias, and nitric oxide (NO)-inducing properties. The antioxidant and aldose reductase inhibitory activities of berberine is useful in alleviating diabetic nephropathy [83]. Berberine specifically binds with DNA to inhibit replication, which confers its cytotoxicity and anticancer properties [28]. Moreover, the low toxicity of berberine makes it a potent future antidiabetic and antiproliferative agent. Berberine production has also been reported from endophytic fungi *Alternaria* sp. and *Fusarium solani* isolated from *Phellodendron amurense* and *Coscinium fenestratum*, respectively [27,29].

#### 2.1.3. Camptothecin

Camptothecin (CPT), a potent anticancer quinoline indole alkaloid, was first isolated from the bark of the *Camptotheca acuminata* in 1966, but it was also produced by some other plant species, including *Miquelia dentata*, *Nothapodytes nimmoniana*, and *Ophiorrhiza* [84]. Inadequate water solubility and high toxicity are two limiting factors for the application of CPT as an anticancer agent. However, its two derivatives 10-hydroxycamptothecin (HCPT), and 9-methoxycamptothecin (MCPT) retain the same medicinal efficacy without above limitations [36]. CPT and HCPT reversibly stabilize the Top1–dsDNA complex by selectively inhibiting eukaryotic topoisomerase I (TopI) activity. In virtue of this, CPT derivatives are currently being used extensively as precursor compounds for efficient broad-spectrum anticancer drugs irinotecan, topotecan, and belotecan [36,85]. Recently, a chemically bespoke camptothecin–antibody drug conjugate named traztuzumabderuxtecan (Enhertu^®^) has also been approved by the US Food and Drug Administration (FDA) [9]. Puri et al. in 2005 and Rehman et al. in 2008 isolated the camptothecin-producing potential endophytic fungi *Entrophospora infrequens* and *Neurospora* sp. respectively from the inner bark of *Nothapodytes foetida* [31,33]. Again, endophytic fungus *F. solani* isolated from *C. acuminata* was found to produce CPT, HCPT, and MCPT [36]. Endophytic fungus *F. solani* isolated from *Apodytes dimidiata* in the Western Ghats, India also yielded camptothecin [39]. Three fungal species, *Alternaria alternata*, *Fomitopsis* sp., and *Phomopsis* sp., isolated from fruits of *M. dentata*, were found as prominent CPT producers [41]. In another bioprospection study, 161 fungal endophytes from *C. acuminata* were screened for CPT production in which *Botryosphaeria dothidea* was found as a prominent producer of MCPT [40]. Two camptothecin-producing fungi, *Trichoderma atroviride* and *Aspergillus* sp., were also isolated from *C. acuminata* [42,85]. CPT-producing endophytic fungi have also been isolated from *N. nimmoniana* [37,86]. We found a total of 22 CPT-producing endophytic fungal species from five different host plant species, as listed in Table 1. These findings suggested that the endophytic fungi could be a future alternative source of not only CPT but also of its safer and more efficient analogues.

#### 2.1.4. Capsaicin

Capsaicin, a spicy alkaloid of red pepper *Capsicum annuum* first crystallized in 1878, has antilithogenic, anti-inflammatory, thermogenic, gastro-stimulatory, antidiabetic, cardioprotective, and anticancer attributes [87]. Capsaicin selectively binds to calcium channel protein targeting transient receptor potential vanilloid 1 (TRPV1) expressed by nociceptors and lowers its opening threshold, resulting in nociceptor depolarization. That is why capsaicin is linked to the sensation of heat and pain as well as obesity regulation via increased thermogenesis. Capsaicin decreases glucose tolerance by inhibiting adipose tissue inflammatory responses via decreasing adipose tissue macrophages and levels of inflammatory adipocytokines like tumor necrosis factor alpha (TNF-α), monocyte chemoattractant protein (MCP)-1, interleukin (IL)-6, and leptin. It also induces the TRPV1-dependent secretion of insulin and antihyperglycemic hormone glucagon. The potential beneficial effects of capsaicin on cardiovascular and gastroprotective systems are exhibited through the TRPV1-mediated release of neurotransmitter calcitonin gene related peptide (CGRP). Capsaicin exerts its anticancer activity via the activation of cAMP-activated protein kinase, peroxisome proliferator-activated receptor gamma (PPARγ)-induced apoptosis, down-regulation of signal transducer and activator of transcription 3 (STAT3) target gene B-cell lymphoma 2 (Bcl-2), cell-cycle arrest by inhibiting cyclin-dependent kinases (CDK2, CDK4 and CDK6), modulation of the human epithelial growth factor receptor 2 (HER2) pathway and p27 expression, down-regulation of p38mitogen-activated protein kinase (MAPK), protein kinase B (PKB or AKT), and focal adhesion kinase (FAK) activation, and degradation of hypoxia inducible factor 1α [88]. An endophytic fungal strain *A. alternata* isolated from fruits of *C. annuum* has also been found to produce capsaicin [44].

#### 2.1.5. Homoharringtonine (HHT)

Valuable anticancer alkaloid homoharringtonine (HHT) for the first time has been isolated from the bark and leaves of threatened medicinal tree *Cephalotaxus harringtonia* [89]. It acts as a translation inhibitor during G_1_ and G_2_ phases of cell division. In 2012, homoharringtonine was approved for the treatment of chronic myeloid leukemia under generic name omacetaxine mepeosuccinate by the Food and Drug Administration of the USA [90]. Hu et al. in 2016 screened 213 fungal strains isolated from the bark of *Cephalotaxus hainanensis* for the HHT biosynthesis ability and found that *Alternaria tenuissima* was a stable HHT-producing endophyte [45].

#### 2.1.6. Huperzine A

A tropical medicinal moss *Huperzia serrata* is clinically used for the treatment of Alzheimer’s disease [54]. Its biologically active alkaloid huperzine A (HupA) acts as a strong acetylcholinesterase inhibitor (AChEI), which is a class of medication that improves the level of neurotransmitters in the brain and is hoped to be a potential treatment for Alzheimer’s disease. Li et al. in 2007 first isolated an HupA-producing endophytic fungus *Acremonium* from *H. serrata* [46]. For now, 12 different HupA-producing endophytic fungal species from *H. serrata* and three from *Phlegmariurus* spp. have been reported by different workers (Table 1). Interestingly, many endophytic fungi have been shown to synthesize novel AChEIs in their metabolite extracts [91].

#### 2.1.7. Peimisine and Imperialine-3β-D-glucoside

*Fritillaria*, a traditional medicinal plant, is among the most widely used antitussive and expectorant drugs. The principal bioactive constituents of Bulbus *Fritillaria cirrhosa* are steroidal alkaloids peimisine and imperialine-3β-D-glucoside [57]. *Fusarium* spp. isolated from *Fritillaria unibracteata* var. *wabensis* have also produced peimisine and imperialine-3β-D-glucoside [58,59].

#### 2.1.8. Piperine

Anti-inflammatory and anticancer alkaloid piperine is found in the fruits of *Piper longum* and *Piper nigrum* and responsible for their pungent taste. Piperine enhances hepatic-oxidized glutathione and decreases renal glutathione concentration and renal glutathione reductase activity, showing its antidiabetic activity. Piperine decreases liver marker enzymes activity, inhibits lipopolysaccharide-induced expression of interferon regulatory factor, reduces the activation of STAT1, and inhibits the release of Th-2-mediated cytokines indicating its anti-inflammatory activity. Piperine expresses its anticancer activity through the following mechanisms: activates caspase-3 and caspase-9, cleaves poly(ADP-ribose) polymerase (PARP), decreases Bcl-2 protein expression and increases Bax protein, reduces the expression of phosphorylated STAT3 and nuclear factor kappa B (NF-kB) transcription factors, blocks extracellular signal-regulated kinase (ERK1/2), p38 MAPK, and AKT signaling pathways, and suppresses epidermal growth factor (EGF)-induced matrix metalloproteinase (MMP)-9 expression [92]. It has bioavailability-enhancing ability for certain drugs and nutrients. It has also been extracted from the cultures of endophytic fungi *Periconia* sp., *C. gloeosporioides*, and *Mycosphaerella* sp. isolated from *Piper* spp. [60,61,62]. Recently, piperine production has also been reported from endophytic *Phomopsis* sp. from *Oryza sativa* [63].

#### 2.1.9. Quinine

The stem bark and roots of the *Cinchona* spp. are well-established sources of quinine. It has been used as the only effective medication for malaria for centuries until the development of synthetic antimalarial drugs in 1940s. Quinine functions as an antimalarial by acting as an intra-erythrocytic schizonticide and also as gametocytocidal for *Plasmodium malariae* and *Plasmodium vivax* but not for *Plasmodium falciparum* [93]. One of the earliest reports regarding the endophytic fungi-based synthesis of quinine was published in 2002 [94]. Maehara and co-workers have found 21 endophytic fungal strains of *Cinchona ledgeriana* positive for quinine synthesis and identified them as strains of *Arthrinium*, *Fomitopsis*, *Diaporthe*, *Penicillium*, *Phomopsis*, and *Schizophyllum* [64]. Similarly, Hidayat et al. reported seven different strains of three *Fusarium* species capable of producing quinine (Table 1) [65].

#### 2.1.10. Rohitukine

Rohitukine, a lead for the semisynthetic potential anticancer drugs flavopiridol (Sanofi-Aventis, Paris, France) and P-276-00 (Piramal Healthcare Ltd., Mumbai, India), is mainly isolated from the bark of *Dysoxylum binectariferum*. However, the removal of bark poses a threat to the survival of the source medicinal plant. Rohitukine exhibits anticancer activity through the up-regulation of p53 and caspase-9 and down-regulation of Bcl-2 protein [95]. In 2012, Kumara and his group isolated an endophyte *Fusarium proliferatum* from *D. binectariferum* that produces host-derived rohitukine [66]. Later, rohitukine-producing other species of *Fusarium* (Table 1) were also recovered from *D. binectariferum* and *Amoora rohituka* [67].

#### 2.1.11. Sanguinarine

Sanguinarine (SA), a toxic benzophenanthridine alkaloid found in the root of *Sanguinaria canadensis* and leaves of *Macleaya cordata*, recently gained attention for its cytotoxic and anticancer activities [68]. It suppresses NF-κB activation and induces a rapid apoptotic response via glutathione depletion, and mitochondrial damage [96]. It exhibits cytotoxicity via affecting the Na^+^-K^+^-ATPase transmembrane protein, which regulates the MAPK pathway, production of reactive oxygen species (ROS), and intracellular calcium level [28]. It also inhibits microtubule polymerization and specifically induces DNA damage in cancer cells. SA has also been produced by endophytic *F. proliferatum* isolated from leaves of *M. cordata* [68].

#### 2.1.12. Solamargine

A well-known medicinal plant *Solanum nigrum* shows anticancer, antioxidant, antimicrobial, hepatoprotective, anti-inflammatory, antipyretic, and diuretic properties due to its flavonoid and steroidal alkaloid contents. Its dominant steroidal alkaloid solamargine has exhibited potent anticancer activity against a wide range of cancer cell lines [97]. Solamargine may induce cell apoptosis via modulating the expression of TNF receptors (TNFRs), down-regulating Bcl-2 and Bcl-xL, increasing caspase-3 activity, and causing DNA damage [98]. Interestingly, an endophytic *A. flavus* isolated from its stem produced more solamargine than the host callus culture [70].

#### 2.1.13. Swainsonine

Swainsonine is an indolizidine alkaloid found in ‘locoweeds’, including *Swainsona canescens, Astragalus,* and *Oxytropis.* It alters glycoprotein processing by inhibiting α-mannosidase and mannosidase II and causes lysosomal storage disease [15]. Research has shown that in *Astragalus*, *Oxytropis,* and *Swainsona* species, swainsonine is produced by endophytic fungi in genera *Embellisia* and *Undifilum* [71,72,74]. Interestingly, in earlier research, plants of *Astragalus* and *Oxytropis* without endophytes were found to be swainsonine-free [99].

#### 2.1.14. Vinblastine and Vincristine

Madagascar periwinkle (*Catharanthus roseus*) is a primary source of well-known anticancer terpenoid indole alkaloids vinblastine and vincristine. They are the second most used class of anticancer drugs in chemotherapy regimens of various malignancies such as acute lymphoblastic leukemia and nephroblastoma [100]. Alkaloid vincristine interferes with spindle formation, intracellular transport, and angiogenesis in tumor cells without affecting normal cells. For the first time in 1998, Guo et al. reported the isolation of vinblastine from an endophytic fungus *Alternaria* sp., residing in *C. roseus* [75]. The endophytic fungi *Fusarium oxysporum*, *Talaromyces radicus,* and *Eutypella* spp. from *C. roseus* produced both vinblastine and vincristine [76,77,78,79].

#### 2.1.15. Vincamine

Indole alkaloid vincamine is one of the most important constituents of *Vinca minor* and *Nerium indicum* (apocynaceae) and is used in treating various cerebrovascular disorders such as hypertension, chronic ischemic stroke, and vascular dementia [101]. In 2011, Yin and Sun reported a vincamine-producing endophyte from the host *V. minor* [81].

### 2.2. Plant-Derived Coumarins (Benzopyrones) from Fungal Endophytes

Coumarins have been routinely employed as herbal remedies since the onset of herbal medicine. It was first isolated as a natural product from seeds of *Dipteryx odorata* (*Coumarouna odorata*) [102]. A total of seven medicinally important specific plant-derived coumarins are produced by fungal endophytes (Table 2).

#### 2.2.1. Bergapten and Meranzin

Furocoumarin bergapten (5-methoxypsoralen) from *Citrus bergamia* and *Balanites aegyptiaca* is a potential photosensitizing drug in the oral photochemotherapy of psoriasis. Bergapten forms a stable combination with pyrimidine bases causing DNA damage and phosphatase and tensin homolog (PTEN)-mediated induced autophagy, indicating anticancer activity [119]. Meranzin exhibits an antidepressant effect through regulation of the α2-adrenoceptor [120]. Meranzin along with bergapten is also found in grapefruit peels [121]. Both of the compounds are also produced by endophytic fungi *Penicillium* sp., *Botryodiplodia theobromae*, and *Alternaria brassicae* [103,104].

#### 2.2.2. Isofraxidin

Isofraxidin is a coumarin compound produced in the Siberian ginseng (*Acanthopanax senticosus* or *Eleutherococcus senticosus*) and *Apium graveolens*. Isofraxidin mainly regulates lipid metabolism and protects from related disorders by reducing triglyceride accumulation, TNF-α release, and ROS activation, enhancing the phosphorylation of AMPKα and acetyl coenzyme A carboxylase (ACC). It also reduces hepatic expression of fatty acid synthase (FAS) and 3-hydroxyl-3-methylglutaryl-CoA synthase 2 (HMGC), inhibiting lipogenesis. Additionally, isofraxidin shows anti-inflammatory activity by significantly depleting infiltrating inflammatory cells (F4/80+ Kupffer cells, and CD68+ macrophages) and inflammatory cytokines (TNF-α and IL-6) in liver cells. Moreover, the anti-inflammatory activity of isofraxidin is correlated with the down-regulation of toll-like receptor 4 (TLR4) and NF-κB expression [122]. Isofraxidin bioactivity as a potent hyperpigmentation agent is exerted by increased melanin synthesis via stimulated tyrosinase activity, increased expression of tyrosinase, and melanogenesis regulator microphthalmia-associated transcription factor (MITF) in melanocytes [123]. The cytotoxic effects of isofraxidin on cancer cells is exerted via inhibition of AKT kinase and increase in caspase-3, caspase-9, and Bax/Bcl-2 levels. Isofraxidin has also shown anti-hypertension effects via inhibiting the activity of angiotensin I converting enzyme (ACE). Isofraxidin protects axons and dendrites against amyloid β (Aβ 25–35) and inhibits neuron-degenerating enzyme monoamine oxidase B [124].

#### 2.2.3. Marmesin

Marmesin (furanocoumarins) was first reported from fruits of *Ammi majus* and later from *Balanites aegyptiaca*, which is a folkloric medicinal plant with purgative, antihelmintic, and antisyphilitic properties [119,125]. It is also synthesized by endophytic *Fusarium* sp. isolated from a mangrove plant [107].

#### 2.2.4. Mellein

Dihydroisocoumarin mellein derives its name from a strain of *Aspergillus melleus*, which is the first reported source of mellein [126]. Later, this compound was found in plants such as *Moringa* and *Stevia* [127,128]. Mellein has exhibited antimicrobial and anti-schistosomiasis activities [114,115]. Mellein has been reported from endophytic fungal species *Septoria nodorum* in 1995 by Findlay et al. followed by dozens of similar reports as listed in Table 2 [108].

#### 2.2.5. Scopoletin and Umbelliferone

Scopoletin (6-methoxy-7-hydroxycoumarin) is a coumarin with antifungal, anti-acetylcholinesterase (AChE), and antitumor properties. Scopoletin inhibits cancer cell proliferation by inducing apoptosis via reducing the protein content and decreasing the acid phosphatase (ACP) activity level [129,130]. Umbelliferone (7-hydroxycoumarin), distributed within the Rutaceae and Apiaceae (Umbelliferae) families, is a fluorescing compound and used as a sunscreen agent. It shows antioxidant, anti-inflammatory, anti-hyperglycemic, anti-tumor, and antimicrobial activities. Umbelliferone exhibits anticancer activity via inducing apoptosis and cell cycle arrest [131].

### 2.3. Plant-Derived Flavonoids from Fungal Endophytes

Flavonoids are pigments of edible plants consisting of two benzene rings at either side of a three-carbon ring. Multiple substitutions in this basic structure produce several classes of derivatives, such as flavones, isoflavones, flavonols, flavanones, catechins, and anthocyanins. We found 12 different biologically active plant-derived flavonoids (Table 3) recovered from fungal endophytes and important flavonoids are described below.

#### 2.3.1. Apigenin

Apigenin, amply present in *Matricaria* spp. and vegetables, has anti-inflammatory, antioxidant, and anticancer properties. Apigenin inhibits the proliferation of malignant cancer cells causing G_2_-M arrest by inhibition of the mitotic kinase activity of p34^cdc2^ and perturbation of cyclin B1 levels [132,154]. It is also a ligand for the central benzodiazepine receptors exerting anxiolytic and sedative effects [155]. Apigenin activates different anti-inflammatory pathways, including p38/MAPK and phosphatidylinositol 3-kinase (PI3K)/AKT, to exert its anti-inflammatory effect. Further, it prevents the IκB degradation and nuclear translocation of the NF-κB, and reduces cyclooxygenase (COX)-2 activity. Additionally, apigenin up-regulates the expression of anti-oxidant enzymes such as glutathione (GSH)-synthase, catalase, and superoxide dismutase (SOD) to counteract cellular oxidative stress. Its neuroprotective effect is exhibited by the lowering of β-amyloids and restoring the ERK/cyclic AMP response element-binding protein (CREB)/ brain-derived neurotrophic factor (BDNF) pathway [156]. Apigenin also regulates hyperglycemia, thyroid dysfunction, and lipid peroxidation [133]. A fungal endophyte *Chaetomium globosum*, isolated from *Cajanus cajan,* produced apigenin with good antioxidant activities [135]. Its glycosidic derivatives, namely apigenin-5-O-α-L-rhamnopyranosyl-(1→3)-β-D-glucopyranoside and euryanoside (apigenin-5-O-α-L-rhamnopyranosyl-(1→2)-(6″-O-acetyl)-β-Dglucopyranoside), were detected in *Paraconiothyrium variabile*, which is an endophytic fungus in the Japanese plum yew (*Cephalotaxus harringtonia*) from which these compounds had previously been reported [136].

#### 2.3.2. Cajanol

Cajanol (phytoalexin) is an isoflavone from roots of *C. cajan* displaying anticancer, antimicrobial, and antiplasmodial activities. Cajanol arrests the cell cycle in the G_2_/M phase and induces apoptosis via the ROS-mediated mitochondria-dependent pathway [157]. Endophytic strains of *Hypocrea lixii* from roots of *C*. *cajan* have also been reported to produce cajanol in aqueous cultures with anticancer activity [137].

#### 2.3.3. Chrysin

The flavonoid chrysin is found in leaves of *P. incarnata* and synthesized by foliar endophytes *A. alternata*, *Colletotrichum capsici*, and *Colletotrichum taiwanense*. It has shown promising biological activities, including antibacterial, anti-inflammatory, antidiabetic, anxiolytic, hepatoprotective, anti-aging, and anticancer effects [139].

#### 2.3.4. Curcumin

Curcumin is the major active principal of *Curcuma* spp. It shows strong anti-inflammatory and antioxidant activities via the downregulation of COX-2, lipoxygenase, TNF-α, IL-1, -2, -6, -8, and Janus kinases. Curcumin anticancer activity involves cell cycle arrest via inhibition of cyclin D1 and CDK4, and induction of apoptotic signals via the up-regulation of Fas, FasL, and DR_5_ expression, p-53 mediated activation of caspase, and inhibition of TNF-α-induced activation of NF-κB [158]. A recent report has suggested curcumin as a potent epigenetic modulator with activities like inhibition of DNA methyltransferases (DNMTs), regulation of histone acetyltransferases (HATs) and histone deacetylases (HDACs), regulation of microRNAs (miRNA). It also interacts with DNA and transcription factors [159]. Curcumin has been isolated from fungal endophytes *Chaetomium globosum* and an unidentified isolate [140,141].

#### 2.3.5. Kaempferol

Kaempferol is a potent antioxidant, anticancer, cardioprotective, neuroprotective, hepatoprotective, and antidiabetic compound found in fruits and vegetables. It blocks the expression of inflammatory cytokines (IL-1B and TNF-α), COX-2 protein, and inducible NO synthase (iNOS). Kaempferol inhibits various cancer cells by arresting cell cycle at the G_2_/M phase, targeting several signaling pathways (MAPK/ERK and PI3K/AKT) that are essential for the survival of cancer cells and modulating expression of epithelial-mesenchymal transition (EMT)-related markers. It prevents the EGF-induced activation of activator protein 1 (AP-1) and NF-κB, and phosphorylation of AKT. It enhances cyclin-dependent kinase inhibitor 1A (CDKN1A) levels via the reduced expression of c-Myc and enhanced level of p53 protein [160,161]. Kaempferol is thereby used as a potent chemopreventive agent in cancer treatment. Endophytic *Fusarium chlamydosporum* as well as its host *Tylophora indica* both can produce keampferol [142]. It is also produced by some other endophytes (Table 3).

#### 2.3.6. Luteolin

Luteolin is a plant metabolite with reputed antioxidant, anti-inflammatory, anticancer, and antidiabetic properties. Its anticancer property is manifested via cell cycle arrest in S phase, modulation in ROS levels, inhibition of topoisomerases type I and II, reduction of NF-kB and AP-1 activity, stabilization of p53, and inhibition of PI3K, STAT3, insulin-like growth factor 1 receptor (IGF1R), and HER2 [162]. Luteolin is a better inhibitor of alpha-glucosidase than the widely prescribed drug acarbose, suggesting its role in reducing high blood sugar levels [163]. It has also been reported as a secondary metabolite of some endophytic fungal strains (Table 3).

#### 2.3.7. Quercetin

Quercetin is a red pigment with antioxidant, anti-inflammatory, anticancer, antiviral, antidiabetic, cardiovascular, and neuroprotective properties that is widely distributed in plants. Quercetin causes cell cycle arrest in the S phase and activates apoptosis in cancer cells [164]. Quercetin along with vitamin C may be used for the prevention of severe acute respiratory syndrome coronavirus-2 (SARS-CoV-2/COVID-19) in high-risk populations [165]. Its antidiabetic activity is exhibited by induced insulin sensitivity and glucose metabolism. The cardiovascular effects of quercetin are exerted by its inhibitory effect on the angiotensin-converting enzyme and by activating Na+-K+-2Cl−cotransporter 1 (NKCC1) in renal epithelial cells [166]. The mechanism behind the neuroprotective effect of quercetin may involve mitigating oxidative stress via induction of nuclear erythroid 2-related factor 2 (Nrf2)/ antioxidant response element (ARE) and antioxidant paraoxonase 2 (PON2) [167]. Recently, three quercetin compounds were extracted from the endophytic fungus *Nigrospora oryzae* isolated from leaves of the Nigerian mistletoe *Loranthus micranthus* [146].

#### 2.3.8. Rutin

Rutin, a glycoside of the flavonoid quercetin with powerful antioxidant, anti-inflammatory, anticancer, and promising neuroprotective properties is found in vegetables and fruits. The anti-inflammatory and antioxidant activities of rutin involves inhibition of expression of COX-2 and iNOS via inhibition of p38 MAPK and c-Jun N-terminal kinase (JNK). Rutin decreases Bcl-2 expression, Bcl-2/Bax ratio, MYCN mRNA levels, and the secretion of TNF-α, demonstrating its anticancer property [168]. Its neuroprotective mechanisms include reduction of pro-inflammatory cytokines, improved antioxidant enzyme activities, activation of MAPK cascade, up-regulation of the ion transport and antiapoptotic genes, and restoration of the activities of mitochondrial complex enzymes [169]. Rutin has also been produced by several endophytic fungi, as listed in Table 3.

#### 2.3.9. Silymarin

Silymarin, a bioactive natural compound found in fruits of milk thistle (*Silybum marianum*), has cardioprotective, hepatoprotective, antioxidant, immunomodulatory, anti-inflammatory, antihepatitic, and antimetastatic activities [170]. The hepatoprotective property of silymarin is accomplished via an increase in glutathione level, inhibition of lipid peroxidation, activation of antioxidant defense, and translational activities in hepatic cells [171]. Its anticancer activity is related to the modulation of NF-kB, suppression of EGFR-MAPK/ERK1/2 and IGF1R signaling, up-regulation of tumor-suppressor genes p53 and p21^CIP1^. Similarly, its antiangiogenic activity is linked to suppression of both vascular endothelial growth factor (VEGF) and MMP-2 [172,173]. Endophytic silymarin for the first time has been reported from the strains of *Aspergillus iizukae* isolated from the leaves and stems of *S. marianum* [152].

#### 2.3.10. Vitexin

C-glycosyl flavonoid vitexin has recently received increased attention due to its wide range of pharmacological effects including anticancer (as hypoxia inducible factor (HIF)-1α inhibitor), analgesic (via targeting TRPV1), antioxidant, hypotensive, neuroprotective, and antidiabetic effects [174]. It is synthesized by plants such as *C. cajan*, *Ficus deltoidea*, *Passiflora incarnata, Vitex agnus-castus,* and endophytic fungi such as *Colletotrichum* sp. from *G*. *biloba* [134] and *Dichotomopilus funicola* of *C. cajan* [153].

### 2.4. Plant-Derived Lignans from Fungal Endophytes

Lignans are secondary metabolites with a plethora of biological activities, making them noteworthy in several lines of research. Out of a total of seven medicinally important plant-derived lignans that have been secreted by endophytes (Table 4), one is described below.

#### Podophyllotoxin

Podophyllotoxin (podofilox), an aryl tetralin lactone lignan of medicinal plant *Podophyllum* sp., is an important anticancer and antiviral agent. It is also found in *Diphylleia*, *Dysosma,* and *Juniperus.* It has been used as the lead for the chemical synthesis of the many useful anticancer drugs such as etoposide, teniposide, and etopophos phosphate [184,185]. Podophyllotoxin is an anti-tubulin agent that destabilizes microtubules. Its derivatives inhibit the topoisomerase II enzyme, which is required to unwind the double helix of DNA, preventing mitosis in late S/early G_2_ phase [192]. For the first time, Yang et al. in 2003 reported the podophyllotoxin producing endophytic fungi (Table 4) from *P. hexandrum*, *Diphylleia sinensis,* and *Dysosma veitchii* [178]. Puri et al. in 2006 reported a fungal endophyte *Trametes hirsuta* from rhizomes of *P. hexandrum* that efficiently produces podophyllotoxin and other related aryl tetralin lignans with potent anticancer properties [182]. Later, several other endophytic fungi such as *Phialocephala fortinii* isolated from the rhizomes of *Podophyllum peltatum*, *Alternaria* sp. isolated from *Juniperus vulgaris,* and *P. hexandrum*, *F. oxysporum* isolated from *Juniperus recurva,* and *A. fumigatus* isolated from *Juniperus communis* have been reported as alternative sources for podophyllotoxin [180,181,183,184,185]. Recently, *A. tenuissima,* a fungal endophyte from the roots of *Podophyllum emodi*, and *Fusarium* sp. from *Dysosma versipellis* showed the presence of podophyllotoxin in their secondary metabolite analysis [188,189]. In total, podophyllotoxin has been isolated from 17 endophytic fungal species collected from 10 different host plant species, as listed in Table 4.

### 2.5. Plant-Derived Saponins from Fungal Endophytes

Saponins are known to occur in many taxonomically unrelated plants, but there is evidence that they are also produced by endophytic fungi (Table 5). We found three specific and several other plant-derived saponins that have been reported from endophytic fungi.

#### Diosgenin

The anti-inflammatory and anticancer agent diosgenin is primarily obtained from *Dioscorea zingiberensis*. Its anti-inflammatory activity is exerted via reduction in the levels of several inflammatory mediators, including NO and IL-1 and -6, inhibition of the MAPK/AKT/NF-κB signaling pathway, and ROS production. Diosgenin anticancer effects have been linked to p53 activation, immune modulation, cell cycle arrest, modulation of caspase-3 activity, and activation of STAT3 signaling pathway [210]. Considering the depleting natural populations and requirement of a long period of rhizome maturation of its primary source *Dioscorea zingiberensis*, endophytes might be suitable alternatives to produce diosgenin. Zhou et al. in 2004 first reported *Paecilomyces* sp. residing in *Paris polyphylla* var. *yunnanensis* as an adiosgenin-producing endophytic fungus [193]. Later, an endophytic strain of *Fusarium* sp. from *Dioscorea nipponica* was also been reported for enhanced production of diosgenin in its liquid cultures when supplemented with the rhizome extract of its host plant [195].

### 2.6. Plant-Derived Terpenes from Fungal Endophytes

Table 6 lists 17 specific plant-derived terpenes produced by fungal endophytes with some of them detailed below.

#### 2.6.1. Artemisinin

Asian plant *Artemisia annua* (sweet wormwood) has been in use for the treatment of fever since more than 2000 years. In 1971, Artemisinin, a sesquiterpene lactone with endoperoxide trioxane moiety, was isolated from the *A. annua* as its active antimalarial principle by Tu Youyou [296]. According to a WHO report, over 2010–2017, about 2.74 billion artemisinin-based combination therapies (ACTs) have been administered globally [297]. Growing evidence revealed that artemisinin and its derivatives have many more biological activities including anti-inflammatory, immunoregulatory, and anticancer activities without any risk of drug-resistant development [298]. Its antimalarial parasite activity is mediated by ROS generation, causing protein damage and compromising parasite proteasome function, inducing the endoplasmic reticulum (ER) stress response [299,300]. Iron (heme), which is a prerequisite for cancer cells multiplication, also activates an endoperoxide bond of artemisinin, creating cytotoxic/cancer-killing carbon-centered free radicals. As an alternative source, Huang et al. isolated artemisinin from an anonymous fungal isolate of *Artemisia indica* [216].

#### 2.6.2. Bilobalide and Ginkgolides

Bilobalide and ginkgolides, two main terpenoids found in the leaves and bark of *G. biloba*, are accountable for the therapeutic implication of its whole extract [301]. Ginkgo products, including EGb-761 registered as a phytomedicine in Europe, are now among the best-selling drugs in the world with US$ 1.26 billion worldwide sales in 2012 [302]. Widely consumed bilobalide (sesquiterpene) has neuroprotective, anti-inflammatory, and analgesic potential and inhibits the diffuse pneumonia caused by *Pneumocystis carinii* [303,304]. Bilobalide has recently been found to be an antagonistic allosteric modulator of the γ-aminobutyric acid A receptors (GABA_A_Rs), linking its role in improving cognitive and memory functioning domain in impaired persons [305]. Recently, *Pestalotiopsis uvicola*, a foliar endophyte of *G. biloba*, has been reported to produce bilobalide [219]. Similarly, ginkgolides are considered as possible drugs based on their key antagonistic effects on the platelet-activating factor (PAF), neuroprotective effects, and protective effects in cardio-cerebral ischemia reperfusion injuries mediated via regulation of TNF-related weak inducer of apoptosis (TWEAK)/ fibroblast growth factor-inducible molecule 14 (Fn14) signaling pathway [301,306,307]. Ginkgolides have also been found in the fermentation products of an endophytic strain of *F. oxysporum* recovered from the root bark of G. *biloba* [226].

#### 2.6.3. Paclitaxel

Paclitaxel (PTX) is a highly functionalized diterpenoid taxane family compound with a four-membered oxetane ring and a C-13 ester side chain. It is used as a basic chemotherapy drug to treat several cancer types and was first extracted from medicinal plant Pacific yew (*Taxus brevifolia*) in 1971 [308]. Paclitaxel binds to the tubulin protein of mitotic spindles, making them nonfunctional. The stabilization of microtubules arrest mitosis in the M phase causes the reversal of cell cycle to the G_0_ phase and induces apoptosis [309]. Two decades after the discovery of paclitaxel ‘taxol’, the US FDA approved it for treating ovarian cancer in 1992 with its commercial sales reaching over $3 billion in 2004 [308]. *T. andreanae* from *Taxus* spp. was the very first endophyte reported to produce paclitaxel, taxol [11]. The above revolutionary discovery was followed by similar findings from 83 different endophytic fungal species isolated from 35 different host plant species including *Taxus* and non-*Taxus* species, as listed in Table 6.

#### 2.6.4. Toosendanin (TSN)

Triterpenoid toosendanin (TSN) is a main bioactive component of fruits and bark of traditional anthelmintic and insecticidal plants *Melia azedarach* and *Melia toosendan*. Toosendanin has antibotulinum (inhibits the botulinum neurotoxin interaction with the SNARE protein), anti-influenza (alters nuclear localization of viral polymerase PA protein), anticancer, anti-inflammatory, and analgesic (selective presynaptic blocker) efficacy [310,311]. Possible actions of TSN as an antitumor drug against a variety of cancer types involve inhibition of STAT3, an emerging target for cancer therapy, induction of estrogen receptor β (ERβ) and p53 proteins, and activation of the mitochondrial apoptotic pathway [312,313,314]. Three unidentified endophytic fungal strains in *M*. *azedarach* have been reported to produce toosendanin [291,292].

#### 2.6.5. Xanthatin

Xanthatin, a natural sesquiterpene lactone of *Xanthium* spp., has significant antimicrobial, trypanocidal, and antitumor activities. Xanthatin exerts its trypanocidal activity by inhibiting both prostaglandin E2 (PGE2) synthesis and 5-lipoxygenase activity, thereby avoiding unwanted inflammation commonly observed in trypanosomiasis. It also permanently inhibits the parasite-specific trypanothione reductase [294]. Xanthatin induces cell cycle arrest at the G_2_/M checkpoint and apoptosis via disrupting the NF-κB pathway [315]. It is also synthesized by endophytic *Paecilomyces* sp. from *Panax ginseng* [295].

### 2.7. Plant-Derived Quinones and Xanthones from Fungal Endophytes

Table 7 lists 20 different plant-derived quinones and xanthones that were reported from fungal endophytes with some important quinones described below.

#### 2.7.1. Hypericin

Hypericin (naphthodianthrone) is a *Hypericum perforatum*-derived antidepressive, antineoplastic, antitumor, antiviral, and photosensitizer compound. Hypericin exerts its antidepressant activity by the inhibition of serotonin, norepinephrine, and dopamine reuptake, increases in IL-6 activity, and the agonist action of sigma receptors [355]. Due to preferential accumulation in neoplastic cells, hypericin can be used in photodynamic diagnosis as an effective fluorescence marker for tumor detection and visualization. Light-activated hypericin is used as a strong pro-oxidant agent in photodynamic therapy to induce the apoptosis, necrosis, or autophagy of cancer cells due to its high affinity for neoplastic cells [356]. It prevents the uncoating of the HIV by stabilizing its capsid and suppresses the release of reverse transcriptase. Later, endophytic fungi *C. globosum*, *Thielavia subthermophila*, and *Epicoccum nigrum* isolated from *H. perforatum* were also found to produce hypericin [324,326,327].

#### 2.7.2. Pachybasin

Pachybasin (anthraquinone) with antimicrobial and antiviral properties was isolated from *Digitalis lanata* [357]. Later, pachybasin was also isolated from *Phoma sorghina,* an endophyte of *Tithonia diversifolia*, and *Coniothyrium* sp., an endophyte of *Salsola oppostifolia* [316,330].

#### 2.7.3. Pinselin (Cassiollin)

Immunosuppressive and anticancer xanthone pinselin was initially characterized from a strain of *Penicillium amarum*, but later found to be identical to cassiollin reported from *Cassia occidentalis* [336]. Later, plant-derived pinselin was reported from endophytic *Phomopsis* sp. isolated from *P. polyphylla* var. *yunnanensis*, *Aspergillus sydowii* isolated from the liverwort *Scapania ciliata*, and *Penicillium* sp. isolated from the leaves of *Sonneratia apetala* [337,338,340].

#### 2.7.4. Plumbagin and Shikonin

Plumbagin and shikonin are anticancer naphthoquinones found in *Plumbago* and *Lithospermum*, respectively. Both can induce in vitro mammalian topoisomerase II-mediated DNA cleavage [358]. The mechanisms underlying the potential antitumor effects of plumbagin involve increased oxidative stress, caspase activity, loss of mitochondrial membrane potential, induction of cytochrome c release, FasL expression, and high Bax levels via activation of the JNK pathway, down-regulation of expression of NF-κB, suppressed TNF-α-induced phosphorylation of p65 and IκB kinase (IKK), degradation of IκBα, and blocking STAT3/ polo-like kinase 1 (PLK1)/AKT signaling [359,360]. Shikonin can induce apoptosis also via ROS generation and the down-regulation of AKT and receptor interacting protein 1 (RIP1)/NF-κB activity [361]. Both the compounds have also been produced by endophytic fungi, as listed in Table 7.

#### 2.7.5. Rhein

*Rheum palmatum* is a highly regarded traditional medicinal plant with cathartic, hepatoprotective, nephroprotective, antimicrobial, anti-inflammatory, anticancer, and antiaging properties. The dominant biologically active constituents in the medicinal roots of *Rheum* are anthraquinones rhein, emodin, and physcion. The hepatoprotective activity of rhein is exerted by its lipid lowering, anti-obesity, anti-inflammatory, and anti-oxidant actions. Rhein also suppresses the expression of alpha-smooth muscle actin (α-SMA) and transforming growth factor-beta (TGF-β), which are indicative of decreased hepatic stellate cell and myofibroblast activation [362]. Nephroprotective properties of rhein arise from its anti-inflammatory action along with the suppression of α-SMA, TGF-β, and fibronectin expression. The anti-inflammatory activity of rhein involves inhibition of the NF-κB pathway, which plays a role in the production of many pro-inflammatory cytokines [363]. The mechanism of rhein anticancer activity involves the inhibition of NF-κB, MAPK, and PI3K/AKT pathways, eventually regulating cell cycle, angiogenesis, and apoptosis [364,365]. Endophytic *F. solani* from the roots of *R. palmatum* also produced the host-derived compounds rhein and emodin [322].

#### 2.7.6. Tanshinones

Diterpenoid quinine metabolite tanshinones (tanshinone I, tanshinone IIA, tanshinone IIB, isotanshinone I, and cryptotanshinone), found in the roots of *Salvia* spp., are considered to be potent anticancer, antiatherosclerosis, antihypertensive, and neuroprotective agents. Tanshinones’ antitumor mechanism involves the inhibition of DNA duplication, cell cycle arrest, regulation of oxidative stress, and reduction of the mitochondrial membrane potential and PTEN-mediated inhibition of the PI3K/AKT pathway to induce apoptosis. Tanshinone I inhibits tumor angiogenesis by the phosphorylation of STAT3 at Tyr705 and hypoxia-induced HIF-1α accumulation in neoplastic cells. The cardiovascular protective effect of tanshinones is exerted by the inhibition of myocardial apoptosis, cardiac fibrosis, atherosclerosis, oxidized low-density lipoprotein (ox-LDL) uptake, thrombin activation, and thrombosis. Tanshinones exhibit significant neuroprotective effects in various neurodegenerative diseases by selectively suppressing pro-inflammatory gene expression in activated microglia, protecting neurons from the neurotoxicity of Aβ, and down-regulating the expression of phosphorylated tau [366]. Endophytic fungi *Phoma glomerata* and *Alternaria* sp. residing in the roots of *Salvia miltiorrhiza* produced tanshinones [352,353]. It has also been secreted by endophytic fungi from *Panex* (Table 7). Interestingly, elicitors from the endophytic fungi *T. atroviride* and *C. globosum* promoted the biosynthesis of tanshinones via enhanced expression of related genes in hairy roots of *S. miltiorrhiza* [367,368].

### 2.8. Miscellaneous Plant-Derived Compounds from Fungal Endophytes

In this section, we grouped diverse classes of compounds such as phenolics, phytoalexins and acids with a total number reaching up to 17 (Table 8).

#### 2.8.1. Cajaninstilbene Acid (CSA)

The major active constituent of leaves of therapeutic pigeon pea extract is cajaninstilbene acid (CSA), which is a low-molecular weight compound containing two benzene rings joined by a molecule of ethylene. Pharmacological studies have shown that CSA exhibits antioxidant, anti-inflammatory, analgesic, and neuroprotective effects. Its cytoprotective effects against oxidative stress is exhibited by inducing the Nrf2-dependent antioxidant pathway and gene expression of heme oxygenase-1 (HO-1), NAD(P)H quinone oxidoreductase 1 (NQO1), and glutamate–cysteine ligase modifier subunits by activation of PI3K/AKT, ERK, and JNK signaling pathways [394]. The anti-inflammatory activity of CSA is associated with the inhibition of NF-κB and MAPK pathways [395]. In a study, CSA attenuated the impairment of learning and memory induced by Aβ (1–42) oligomers by stimulating Aβ clearance and inhibiting microglial activation and astrocyte reactivity in the hippocampus. It also decreased the high levels of Glu but increased the low levels of GABA. In addition, CSA inhibited the excessive expression of GluN2B-containing N-methyl-D-aspartate receptors (NMDARs) and up-regulated the downstream protein kinase A (PKA)/CREB/ BDNF/ tropomyosin receptor kinases (TrkB) signaling pathway. The above findings imply that CSA could be a potential neuroprotective agent at the early stage of Alzheimer’s disease [396]. CSA has also been produced by pigeon pea endophytic fungi *Alternaria*, *Fusarium* spp., and *Neonectria macrodidym* [137,376].

#### 2.8.2. Digoxin

The glycoside digoxin from *Digitalis* spp. has been reported to be cardiotonic and is widely used in the treatment of various heart disorders such as atrial fibrillation, atrial flutter, and heart failure. Digoxin induces an increase in intracellular sodium followed by calcium in the heart by reversibly inhibiting the activity of the myocardial Na+-K+-ATPase pump, leading to an increased force of myocardial contraction and cardiac output. By stimulating the parasympathetic nerve, it slows electrical conduction in the AV node by increasing the refractory period of cardiac myocytes; therefore, it decreases the ventricular response and heart rate. Overall, the stroke volume is increased while the heart rate is decreased, resulting in a net increase in blood pressure [397]. Crude extracts of fungal cultures isolated from *Digitalis lanata* also showed the production of digoxin [382].

#### 2.8.3. Forskolin (Coleonol)

The roots of Indian Coleus (*Coleus forskohlii*) contain a biologically active labdane diterpene compound forskolin with antiglaucoma, anti-HIV, and antitumor activities. Other approved and potential applications of forskolin range from the treatment of hypertension and heart failure to lipolysis and body weight control [398]. Forskolin activates a variety of adenylate cyclase systems to increase the cellular concentrations of cyclic AMP, which is an important second messenger necessary to elicit cAMP-dependent physiological responses [399]. An endophytic fungus *Rhizoctonia bataticola* isolated from *C. forskohlii* was found to synthesize forskolin [383].

#### 2.8.4. Salidroside and p-Tyrosol (Aglycone of Salidroside)

*Rhodiola rosea*, a traditional medicinal herb used as stimulant and antidepressant, has great pharmaceutical value including antioxidant, antihypoxic, adaptogenic, cardiovascular, and neuroprotective properties. Its active principals are phenolics (salidroside and p-tyrosol) and glycosides (rosavins) [400]. Salidroside and p-tyrosol inhibit the hypoxia-induced endocytosis of pulmonary Na,K-ATPase via the inhibition of the ROS– AMPK–protein kinase Cζ (PKCζ) pathway, signifying the use of *Rhodiola* as a popular folk medicine for high-altitude illness [401]. The mechanisms underlying the potential neuroprotective effects of salidroside involve the regulation of oxidative stress response, inflammation, apoptosis, hypothalamus–pituitary–adrenal axis, neurotransmission, neural regeneration, and the cholinergic system [402]. Cui et al. isolated four endophytic fungi from different species of *Rhodiola* that could produce salidroside and p-tyrosol and characterized *P. fortinii* as the most capable and stable producer [392].

## 3. Avenues and Challenges in Application of Endophyte as Alternative Sources of Plant-Derived Natural Compounds

The success of natural products in drug discovery lies in their enormous structural diversity, diverse pharmacological activities, safety, and inherent binding capacity with other biomolecules [2,9,298]. Reports regarding the biosynthesis of plant-derived natural compounds from endophytic fungi coupled with recent dynamic progress in fermentation, extraction, purification, characterization, and bioassay techniques have enabled us to rapidly characterize valuable novel natural products and access earlier inaccessible endophytic resource [403,404]. Generally, the fermentation process for fungi is short, simple, and economically feasible with a great degree of flexibility for modulation by feeding precursors, elicitors, special enzymes, and modifiers for the efficient enhanced production of bioactive compounds. Endophytes can uniquely biotransform original plant-derived bioactive compounds to their more efficient derivatives, leading to structural and functional diversification [77,136,146]. These studies have evidenced the incredible manipulability of fungal secondary metabolism. There are cases where endophytes up-regulated the synthesis of host compounds and the expression of related genes in the plant host. Hence, each report of the biosynthesis of plant-derived natural compounds from fungal endophytes clearly presents a hopeful way for the efficient and specific production of valuable bioactive natural compounds using endophytes as stable and smart “bio-laboratories”.

However, this approach needs to overcome certain challenges. First, there is an ongoing search for highly productive endophytic fungi for desired plant-derived compounds followed by their strain improvement through epigenetic modulations, mutations, and genetic engineering to make them suitable for industrial applications. Furthermore, we need to elucidate the complete biosynthesis route including all the enzymes and related genes involved through ‘omics’—genomics, transcriptomics, proteomics, and metabolomics—to regulate and manipulate the biosynthesis process for improved productivity [405,406,407]. Alternatively, the identified biosynthetic pathway of the bioactive compounds can be assembled and mimicked in convenient systems, offering an approach to produce target compounds with ease. Second, we need to know more about the roles of host plant–endophyte interactions, requirements of plant niche, and identities of specific signals/elicitors in the synthesis and induction of host-derived natural compounds by endophytes under the OSMAC strategy to overcome the problem of low yield and attenuation, the major challenges for commercial success of this novel approach [406,408,409,410]. The reasons for the attenuation of products have been attributed to the lack of apparent signals/molecules arising from host–endophyte and/or endophyte–peer endophytes interactions in axenic monocultures, resulting in the switching off of genes [86]. However, characterization of the specific nature of assumed activator signals/molecules remains to be done. Third, this area needs collaborations between scientists working in this area and in the pharmaceutical industry for the successful industrial scale production of pharmaceutically valuable compound/leads [411]. The pharmaceutical industry must prioritize their endeavors toward the endophyte-dependent biosynthesis of plant-derived natural compounds.

## 4. Conclusions

After screening a large spectrum of articles dedicated to endophyte research, natural product drug discovery, combinatorial chemistry, genomics, metabolomics ethnobotany, modern medicine, and multidisciplinary science, we curated 101 specific plant-derived medicinal compounds efficiently biosynthesized by hundreds of endophytic fungi. Nonetheless, the exciting progress that has been made in the field of functional genomics, genome mining and genome scanning, fermentation technology, green combinatorial chemistry, and systems biology might remove the roadblocks in the way of commercial success of this innovative approach [282,412,413]. In conclusion, the pursuit of the idea of endophyte-dependent enhanced in vivo and in vitro production of plant-derived valuable metabolites is of prime importance for the pharmaceutical industries, for the health care systems, and for a “green drug revolution”.

## Figures and Tables

**Table 1:** Plant-derived alkaloids produced by endophytic fungi.

Plant-Derived Alkaloids	Activities/Applications	Plant Source	Endophytic Source	Host Plant	References
Aconitine	Anticancer, anti-inflammatory, anti-neuralgic, cardiotoxic	*Aconitum* spp.	*Cladosporium cladosporioides*	*Aconitum leucostomum*	[26]
Berberine	Antibiotic, antidiabetic, antihypertensive, antiproliferative hepatoprotective, hypolipidemic, vasodilator	*Berberis* spp., *Coscinium fenestratum*, *Hydrastis canadensis*, *Phellodendron amurense*	*Alternaria* sp.	*Phellodendron amurense*	[27]
			*Fusarium solani*	*Coscinium fenestratum*	[28,29]
Caffeine	CNS stimulant	*Coffea* spp., *Theobroma cacao*	Anonymous endophytes	*Osbeckia chinensis,* *Osbeckia stellata*, *Potentilla fulgens*	[30]
Camptothecin	Antitumor	*Camptotheca acuminata*, *Miquelia dentata*, *Nothapodytes nimmoniana*, *Ophiorrhiza* spp.	*Entrophospora infrequens*	*Nothapodytes foetida*	[31]
			*Entrophospora infrequens*	*Nothapodytes foetida*	[32]
			*Neurospora* sp.	*Nothapodytes foetida*	[33]
			*Valsa mali*	*Camptotheca acuminata*	[34]
			*Nodulisporium* sp.	*Nothapodytes foetida*	[35]
			*Fusarium solani*	*Camptotheca acuminata*	[36]
			*Botryosphaeria parva*, *Diaporthe conorum*, *Fusarium oxysporum*, *Fusarium sacchari*, *Fusarium solani*, *Fusarium subglutinans*, *Fusarium verticillioides*, *Galactomyces* sp., *Irpex lacteus*, *Phomopsis* sp., *Fusarium* sp.	*Nothapodytes nimmoniana*	[37]
			*Xylaria* sp.	*Camptotheca acuminata*	[38]
			*Fusarium solani*	*Apodytes dimidiata*	[39]
			*Botryosphaeria dothidea*	*Camptotheca acuminata*	[40]
			*Alternaria alternata*, *Fomitopsis* sp., *Phomopsis* sp.	*Miquelia dentata*	[41]
			*Trichoderma atroviride*	*Camptotheca acuminata*	[42]
			*Aspergillus* sp.	*Camptotheca acuminata*	[36]
			*Fusarium oxysporum*	*Nothapodytes foetida*	[43]
Capsaicin	Anti-inflammatory, gastro-stimulatory	*Capsicum annuum*	*Alternaria alternata*	*Capsicum annuum*	[44]
Homoharringtonine	Anticancer	*Cephalotaxus* spp.	*Alternaria tenuissima*	*Cephalotaxus* sp.	[45]
Huperzine A	Acetylcholinesterase inhibitor, Alzheimer’s treatment	*Huperzia serrata*	*Acremonium* sp.	*Huperzia serrata* (syn. *Lycopodium serratum*)	[46]
			*Blastomyces* sp., *Botrytis* sp.	*Phlegmariurus cryptomerianus*	[47]
			*Penicillium chrysogenum*	*Huperzia serrata*	[48]
			*Shiraia* sp.	*Huperzia serrata*	[49]
			*Cladosporium cladosporioides*	*Huperzia serrata*	[50]
			*Colletotrichum* sp., *Trichoderma* sp.	*Huperzia serrata*	[51]
			*Paecilomyces tenuis*	*Huperzia serrata*	[52]
			*Aspergillus flavus*, *Mycoleptodiscus terrestris*, *Penicillium griseofulvum*	*Huperzia serrata*	[53]
			*Penicillium* sp.	*Huperzia serrata*	[54]
			*Fusarium* sp.	*Phlegmariurus taxifolius*	[55]
			*Fusarium* sp.	*Huperzia serrata*	[56]
Peimisine, Imperialine-3b-D-glucoside	Antiasthmatic, antitumor, expectorant	*Fritillaria* spp.	*Fusarium* sp.	*Fritillaria unibracteata* var*. wabuensis*	[57,58]
			*Fusarium redolens*	*Fritillaria unibracteata* var*. wabuensis*	[59]
Piperine	Anti-inflammatory, anticancer, antimicrobial, antidepressant, hepatoprotective	*Piper longum*, *Piper nigrum*	*Periconia* sp.	*Piper longum*	[60]
			*Colletotrichum gloeosporioides*	*Piper nigrum*	[61]
			*Mycosphaerella* sp.	*Piper nigrum*	[62]
			*Phomopsis* sp.	*Oryza sativa*	[63]
Cinchona alkaloids: Quinine, Quinidine, Cinchonidine, Cinchonine	Antimalarial, antiarrhythmic, analgesic	*Cinchona* spp.	*Arthrinium*, *Fomitopsis*, *Diaporthe*, *Penicillium*, *Phomopsis*, *Schizophyllum*	*Cinchona ledgeriana*	[64]
			*Fusarium incarnatum*, *Fusarium oxysporum* (only quinine and cinchonidine) *Fusarium solani* (only quinine)	*Cinchona calisaya*	[65]
Rohitukine	Anticancer, CDK inhibitor, cytotoxic	*Amoora rohituka* *Dysoxylum binectariferum*,	*Fusarium proliferatum*	*Dysoxylum binectariferum*	[66]
			*Fusarium oxysporum*, *Fusarium solani*	*Dysoxylum binectariferum*	[67]
			*Gibberella fujikuroi*	*Amoora rohituka*	[67]
Sanguinarine	Anticancer, antimicrobial, anti-inflammatory antioxidant, antihelmintic, neuroprotective	*Macleaya cordata*, *Sanguinaria canadensis*	*Fusarium proliferatum*	*Macleaya cordata*	[28,68]
Sipeimine	Antibechic, anti-ulcer	*Fritillaria* spp.	*Cephalosporium corda*	*Fritillaria ussuriensis*	[69]
Solamargine	Anticancer, cytotoxic	*Solanum nigrum*	*Aspergillus flavus*	*Solanum nigrum*	[70]
Swainsonine	Toxicosis in livestock	*Astragalus*, *Oxytropis* spp., *Swainsona canescens*	*Embellisia* sp.	*Astragalus*, *Oxytropis* spp.	[15,71]
			*Undifilum cinereum*, *U. fulvum*	*Astragalus lentiginosus*, *Astragalus mollissimus*	[72]
			*Fusarium tricinctum*	*Oxytropis deflexa*, *Oxytropis kansuensis*	[73]
			*Undifilum* sp.	*Swainsona canescens*	[74]
Vinblastine, Vincristine	Antitumor	*Catharanthus roseus* (syn. *Vinca rosea*)	*Alternaria* sp.	*Catharanthus roseus*	[75]
			*Fusarium oxysporum*	*Catharanthus roseus*	[76]
			*Fusarium oxysporum*	*Catharanthus roseus*	[77]
			*Talaromyces radicus*	*Catharanthus roseus*	[78]
			*Eutypella* spp.	*Catharanthus roseus*	[79]
			*Geomyces* sp.	*Nerium indicum*	[80]
Vincamine	Antihypertensive, vasodilator	*Vinca minor*	Anonymous	*Vinca minor*	[81]

**Table 2:** Plant-derived coumarins produced by endophytic fungi.

Plant-Derived Coumarins	Activities/Applications	Plant Source	Endophytic Source	Host Plant	References
Bergapten, Meranzin	Antioxidant, psoriasis treatment	*Balanites aegyptiaca*, *Citrus bergamia*, Grapefruit peel	*Penicillium* sp.	*Avicennia*	[103]
			*Botryodiplodia theobromae*	*Dracaena draco*	[104]
Isofraxidin	Anticancer, anti-obesity, cardioprotective, neuroprotective, hyper pigmentation	*Acanthopanax senticosus*, *Sarcandra glabra*	*Annulohypoxylon bovei* var. *microspora*	*Cinnamomum* sp.	[105,106]
Marmesin	Anticancer, antihelmintic, antioxidant, antisyphilitic, purgative	*Ammi majus*, *Balanites aegyptiaca*	*Fusarium* sp.	Mangrov*e*	[107]
Mellein	Antibacterial, antifungal, antihepatitis c, larvicidal, phytotoxic	*Alibertia macrophylla*, *Litsea akoensis*, *Garcinia bancana*, *Moringa oleifera*, *Stevia lucida*	*Septoria nodorum*	Conifer	[108]
			*Penicillium janczewskii*	*Prumnopitys andina*	[109]
			*Botryosphaeria mamane*	Anonymous	[110]
			A xylariaceous fungus	*Sapindus saponaria*	[111]
			*Annulohypoxylon bovei* var. *microspora*	*Cinnamomum* sp.	[106]
			*Penicillium* sp., *Xylaria* sp.	*Alibertia macrophylla*, *Piper aduncum*	[112]
			*Annulohypoxylon squamulosum*	*Cinnamomum* sp.	[113]
			*Nigrospora* sp.	*Moringa oleifera*	[114]
			*Arthrinium* (*Apiospora montagnei*)	Anonymous	[115]
			*Xylaria* sp.	*Garcinia* sp.	[116]
			*Pezicula* sp.	*Forsythia viridissima*	[117]
			*Xylaria cubensis*	*Litsea akoensis*	[118]
Scopoletin, Umbelliferone	Antifungal, antioxidant, anti-inflammatory	*Artemisia scoparia*, *Scopolia carniolica* (syn. *Scopolia japonica*), *Viburnum prunifolium*	*Penicillium* sp.	*Avicennia*	[103]

**Table 3:** Plant-derived flavonoids produced by endophytic fungi.

Plant-Derived Flavonoids	Activities/Applications	Plant Source	Endophytic Source	Host Plant	References
Apigenin	Antibacterial, anticancer, antioxidant, antihyperglycaemic, lipid peroxidation, sedative, thyroid dysfunction	*Cajanus cajan*, *Cephalotaxus harringtonia*, *Matricaria chamomilla*, vegetables	*Colletotrichum* sp.	*Ginkgo biloba*	[132,133,134]
			*Chaetomium globosum*	*Cajanus cajan*	[135]
			*Paraconiothyriu mvariabile*	*Cephalotaxus harringtonia*	[136]
Cajanol	Anticancer, antimicrobial, antiplasmodial	*Cajanus cajan*	*Hypocrealixii*	*Cajanus cajan*	[137]
Chalcone	Antibacterial, antifungal, antitumor, anti-inflammatory	*Cleistocalyx operculatus*, Members of Leguminosae, Asteraceae, Moraceae	*Ceriporia lacerata*	*Cleistocalyx operculatus* (syns. *Eugenia operculata*, *Syzygium operculatum*)	[138]
Chrysin	Antiaging, anticonvulsant, antidiabetic, anti-inflammatory, antimicrobial, anxiolytic, hepatoprotective	*Passiflora incarnata*	*Alternaria alternata, Colletotrichum capsici*, *Colletotrichum taiwanense*	*Passiflora incarnata*	[139]
Curcumin	Anti-inflammatory, antioxidant, antitumor	*Curcuma* spp.	*Chaetomium globosum*	*Curcuma wenyujin*	[140]
			Anonymous	*Curcuma wenyujin*	[141]
Kaempferol	Antibacterial, antidiabetic, anti-inflammatory, antioxidant, antitumor	Fruits, vegetables, medicinal herbs	*Annulohypoxylonboveri* var. *microspora*, *Annulohypoxylon squamulosum*	*Cinnamomum* sp.	[106,113]
			*Fusarium chlamydosporum*	*Tylophora indica*	[142]
			*Mucor fragilis*	*Podophyllum hexandrum*	[143]
Luteolin	Anti-inflammatory, antioxidant, immunomodulatory	Fruits, vegetables, medicinal herbs	*Annulohypoxylon boveri* var. *microspora*	*Cinnamomum* sp.	[106]
			*Aspergillus fumigatus*	*Cajanus cajan*	[144]
Quercetin	Anticancer, anti-inflammatory antioxidant	Fruits, vegetables	*Aspergillus nidulans,* *Aspergillus oryzae*	*Ginkgo biloba*	[145]
			*Annulohypoxylon squamulosum*	*Cinnamomum* sp.	[113]
			*Nigrospora oryzae*	*Loranthus micranthus*	[146]
Rotenone	Insecticide, pesticide, piscicide	*Derris elliptica*	*Penicillium* sp.	*Derris elliptica*	[147]
Rutin	Antioxidant, cardioprotective, neuroprotective	*Aegle marmelos* *Ginkgo biloba*, *Nerium oleander*, *Pteris multifida*, fruits, vegetables	Anonymous	*Pteris multifida*	[148]
			*Chaetomium* sp.	*Nerium oleander*	[149]
			*Xylaria* sp.	*Ginkgo biloba*	[150]
			*Aspergillus flavus*	*Aegle marmelos*	[151]
Silymarin	Anticancer, antioxidant, anti-inflammatory, cardioprotective, hepatoprotective	*Silybum marianum*	*Aspergillus iizukae*	*Silybum marianum*	[152]
Vitexin	Antioxidant, antitumor, neuroprotective	*Cajanus cajan*, *Vitex agnus-castus*	*Colletotrichum* sp.	*Ginkgo biloba*	[134]
			*Dichotomopilus funicola*	*Cajanus cajan*	[153]

**Table 4:** Plant-derived lignans produced by endophytic fungi.

Plant-Derived Lignans	Activities/Applications	Plant Source	Endophytic Source	Host Plant	References
Coniferin	Antidiabetic	*Angelica archangelica*, Conifers	Members of xylariaceae	*Angelica archangelica*	[175]
Phillyrin	Antioxidant, antidiabetic, anti-inflammatory, anti-obesity, antipyretic	*Forsythia suspensa*, *Phyllirea*	*Colletotrichum gloeosporioides*	*Forsythia suspensa*	[176,177]
Podophyllotoxin	Antitumor, antivirus	*Diphylleia* sp., *Dysosma* sp., *Juniperus* sp., *Podophyllum* spp.	*Alternaria* sp., *Penicillium* spp.	*Podophyllum hexandrum*	[178]
			*Monilia* sp., *Penicillium* sp.	*Dysosma veitchii*	[178]
			*Penicillium* sp.	*Diphylleia sinensis*	[178]
			*Penicillium implicatum*	*Diphylleia sinensis*	[179]
			*Alternaria* sp.	*Juniperus vulgaris*	[180]
			*Phialocephala fortinii*	*Podophyllum peltatum*	[181]
			*Trametes hirsuta*	*Podophyllum hexandrum*	[182]
			*Alternaria neesex*	*Podophyllum hexandrum*	[183]
			*Fusarium oxysporum*	*Juniperus recurva*	[184]
			*Aspergillus fumigatus*	*Juniperus communis*	[185]
			*Fusarium solani*	*Podophyllum hexandrum*	[186]
			*Mucor fragilis*	*Podophyllum hexandrum*	[143]
			*Phialocephala podophylli*	*Podophyllum peltatum*	[187]
			*Alternaria tenuissima*	*Podophyllum emodi*	[188]
			*Fusarium* sp.	*Dysosma versipellis*	[189]
Sesamin, Syringaresinol, Ketopinoresinol	Antioxidant, anti-inflammatory	*Cinnamomum cassia*	*Annulohypoxylon ilanense*	*Cinnamomum* sp.	[190]
Syringin	Antidiabetic	*Syringa vulgaris*, *Eleutherococcus senticosus*, *Magnolia sieboldii*, *Musa paradisiaca*	Members of xylariaceae	*Syringa vulgaris*	[175,191]

**Table 5:** Plant-derived saponins produced by endophytic fungi.

Plant-Derived Saponins	Activities/Applications	Plant Source	Endophytic Source	Host Plant	References
Diosgenin	Anti-inflammatory, antitumor, cardiovascular protection	*Dioscorea* spp.	*Paecilomyces* sp.	*Paris polyphylla* var. *yunnanensis*	[193]
			*Cephalosporium* sp.	*Paris polyphylla* var. *yunnanensis*	[194]
			*Fusarium* sp.	*Dioscorea nipponica*	[195]
Ginsenoside	Anti-inflammatory, antioxidation, antitumor	*Panax*	*Camarosporium* sp., *Dictyochaeta* sp., *Penicillium* sp.	*Aralia elata*	[196]
			*Aspergillus* sp., *Fusarium* sp., *Verticillium* sp.	*Panax ginseng*	[197]
			*Aspergillus* sp., *Fusarium* sp.	*Panax notoginseng*	[198]
Gymnemagenin	Antidiabetic	*Gymnema sylvestre*	*Penicillium oxalicum*	*Gymnema sylvestre*	[199]
Other saponins	Cardiovascular disease	*Gynostemma pentaphyllum*, *Manilkara zapota*, *Sapindus* sp., *Saponaria* sp.	*Aspergillus niger*, *F. oxysporum*	*Crotalaria pallida*	[200]
			*Alternaria alternata, Aspergillus niger*, *Penicillium* sp.	*Loranthus* sp.	[201]
			*Alternaria alternata*, *Aspergillus flavus*, *Aspergillus niger*, *Colletotrichum gleosporioides*, *Trichoderma* sp.	*Tabebuia argentea*	[202]
			*Aspergillus* sp.	*Salvadora oleoides*	[203]
			*Aspergillus* sp.	*Justicia beddomei*	[204]
			*Cochliobolus lunatus* (anamorph *Curvularia lunata)*	*Boswellia ovalifoliolata*	[205]
			*Monochaetia karstenii* (syn. *Pestalotiopsis maculans*), *Phyllosticta* sp.	*Shorea thumbuggaia*	[205]
			*Aspergillus neoniveus* (syn. *Fennellia nivea*)	*Typhonium divaricatum*	[206]
			*Alternaria alternata*, *Aspergillus flavus*, *Aspergillus niger*, *Cladosporium* sp., *Penicillium* sp., *Phomopsis* sp., *Trichoderma* sp.	*Aegle marmelos*	[207]
			*Aspergillus niger, Aspergillus* sp., *Aspergillus terreus*, *Aspergillus tubingensis*, *Coprinopsis cinerea*, *Curvularia lunata*, *Fusarium* sp.	*Eugenia jambolana*	[208]
			*Aspergillus awamori*, *Colletotrichum gleosporioides*	*Rauwolfia* *serpentina*	[209]

**Table 6:** Plant-derived terpenes produced by endophytic fungi.

Plant-Derived Terpenes	Activities/Applications	Plant Source	Endophytic Source	Host Plant	References
Agathic acid	Abortifacient, anti-inflammatory, anticancer, trypanocidal	*Agathis* spp., *Copaifera* spp., *Juniperus osteosperma*	*Botryosphaeria* sp.	*Maytenus hookeri*	[211,212,213]
			*Bionectria* sp.	*Raphia taedigera*	[214]
			*Fusarium* sp.	*Santalum album*	[215]
Artemisinin	Antimalarial	*Artemisia* spp.	Anonymous	*Artemisia indica*	[216]
Asiaticoside	Antidermatitic, anti-inflammatory, antioxidant, immunomodulatory	*Centella asiatica*	*Colletotrichum gloeosporioides*	*Centella asiatica*	[217]
Azadirachtin	Hepatoprotective, insecticidal	*Azadirachta indica*	*Penicillium* (*Eupenicillium*) *parvum*	*Azadirachta indica*	[218]
Bilobalide	Neuroprotective,	*Ginkgo biloba*	*Pestalotiopsis uvicola*	*Ginkgo biloba*	[219]
Borneol	Antiapoptotic, anti-inflammatory, antioxidant, neuroprotective	*Cinnamomum camphora* var. *borneol*	*Cochliobolus nisikadoi*	*Cinnamomum camphora* var. *borneol*	[220]
Camphor	Antimicrobial, topical skin preparations	*Cinnamomum camphora*	*Nodulisporium* sp.	*Lagerstroemia loudoni*	[221]
Cineole (Eucalyptol)	Antimicrobial, respiratory illness	*Eucalyptus* spp.	*Hypoxylon* sp., *Nodulisporium* sp.	*Persea indica*	[222]
			*Nodulisporium* sp.	*Lagerstroemia loudoni*	[221]
			*Nodulisporium* sp.	*Thelypteris angustifolia*	[223]
			*Nodulisporium* sp.	*Cassia fistula*	[224]
			*Annulohypoxylon* sp.	*Neolitsea pulchella*	[225]
Dihydrocumambrin	Antibacterial, cytotoxic	*Glebionis coronaria* (syn. *Chrysanthemum coronarium*)	*Botryodiplodia theobromae*	*Dracaena draco*	[104]
Ginkgolide	Antiallergic, anti-inflammatory	*Ginkgo biloba*	*Fusarium oxysporum*	*Ginkgo biloba*	[226]
Isocupressic acid	Abortifacient	Conifers	*Botryosphaeria* sp.	*Maytenus hookeri*	[212,213]
Loliolide	Herbivore resistance	*Lolium perenne*	*Annulohypoxylon ilanense*	*Cinnamomum* sp.	[227]
Taxane (other than taxol)	Anticancer	*Taxus* spp.	*Alternaria, Aspergillus*, *Beauveria, Epicoccum*, *Fusarium, Gelasinospora*, *Geotrichum, Phoma*, *Phomopsis*	*Taxus baccata*	[228]
			*Cladosporium langeronii*, *Phomopsis* sp.	*Wollemia nobilis*	[229]
Taxol	Anticancer	*Taxus brevifolia*	*Taxomyces andreanae*	*Taxus brevifolia*	[11]
			*Taxomyces* sp.	*Taxus yunnanensis*	[230]
			*Pestalotiopsis microspora*	*Taxodium distichum*	[231]
			*Alternaria* sp., *Pestalotiopsis microspora*	*Taxus cuspidata*	[232]
			*Fusarium lateritium*, *Monochaetia* sp., *Pestalotia bicilia*	*Taxus baccata*	[232]
			*Pithomyces* sp.	*Taxus sumatrana*	[232]
			*Pestalotiopsis microspora*	*Taxus wallichiana*	[233]
			*Pestalotiopsis guepinii*	*Wollemia nobilis*	[234]
			*Periconia* sp.	*Torreyagrandifolia*	[235]
			*Seimatoantlerium nepalense*	*Taxus wallichiana*	[236]
			*Alternaria* sp., *Pestalosiopsis* sp.	*Ginkgo biloba*	[237]
			*Penicillium raistrickii*	*Taxus brevifolia*	[238]
			*Tubercularia* sp.	*Taxus chinensis* var. *mairei*	[239]
			*Stegolerium kukenani*	*Kukenan tepuis*, *Roraima*	[240]
			*Taxomyces* sp.	*Taxus* sp.	[241]
			*Sporormia minima*, *Trichothecium* sp.	*Taxus wallichiana*	[242]
			*Nodulisporium sylviforme*	*Taxus cuspidata*	[243]
			Anonymous	*Taxus chinensis* var. *mairei*	[244]
			*Botrytis* sp.	*Taxus chinensis* var. *mairei*	[245]
			*Penicillium* sp.	*Taxus yunnanensis*	[246]
			*Fusarium mairei*	*Rhizophora annamalayana*	[247]
			*Phyllosticta* sp.	*Ocimum basilicum*	[248]
			*Alternaria alternata*, *Ectostroma*sp., *Fusarium mairei*, *Ozonium*sp., *Papulaspora* sp.	*Taxus chinensis* var. *mairei*	[249]
			*Fusarium solani*	*Taxus celebica*	[250]
			*Pestalotiopsis pauciseta*	*Cardiospermum helicacabum*	[251]
			*Bartalinia robillardoides*	*Aegle marmelos*	[252]
			*Colletotrichum gloeosporioides*	*Justicia gendarussa*	[253]
			*Fusarium* sp.	*Taxus wallichiana*	[254]
			*Phyllosticta citricarpa*	*Citrus medica*	[255]
			*Phyllosticta melochiae*	*Melochia corchorifolia*	[256]
			*Phyllosticta spinarum*	*Cupressus* sp.	[257]
			*Fusarium arthrosporioides*	*Taxus cuspidata*	[258]
			*Aspergillus fumigatus*	*Podocarpus* sp.	[259]
			*Botryodiplodia theobromae*	*Taxus baccata*	[260]
			*Botrytis* sp.	*Taxus cuspidata*	[261]
			*Fusarium solani*	*Taxus chinensis*	[262]
			*Chaetomella raphigera*	*Terminalia arjuna*	[263]
			*Pestalotiopsis terminaliae*	*Terminalia arjuna*	[264]
			*Phomopsis* sp.	*Ginkgo biloba*	[265]
			*Phomopsis* sp.	*Larix leptolepis*	[265]
			*Phomopsis* sp.	*Taxus cuspidata*	[265]
			*Phyllosticta dioscoreae*	*Hibiscus rosa-sinensis*	[266]
			*Aspergillus* sp., *Ceratobasidium* sp., *Cladosporium tenuissimum*, *Coniothyrium diplodiella*, *Epacris* sp., *Fusarium solani*, *Metarhizium anisopliae*, *Paraconiothyrium brasiliense*, *Pezicula* sp., *Phomopsis* sp. *Sordaria* sp., *Trichoderma* sp., *Xylaria* sp.	*Taxus chinensis*	[267]
			*Mucor rouxianus*	*Taxus chinensis*	[268]
			*Colletotrichum gloeosporioides*	*Plumeria acutifolia*	[269]
			*Gliocladium* sp.	*Taxus baccata*	[270]
			*Pestalotiopsis* sp.	*Catharanthus roseus*	[271]
			*Aspergillus candidus*, *Cladosporium cladosporioides*	*Taxus media*	[272,273]
			*Aspergillus niger* var. *taxi*	*Taxus cuspidata*	[274]
			*Mucor* sp.	*Taxus chinensis* var. *mairei*	[275]
			*Pestalotiopsis neglecta*, *Pestalotiopsis versicolor*	*Taxus cuspidata*	[276]
			*Pestalotiopsis pauciseta*	*Tabebuia pentaphylla*	[277]
			*Lasiodiplodia theobromae*	*Morinda citrifolia*	[278]
			*Acremonium* sp., *Botryosphaeria* sp., *Fusarium* sp., *Gyromitra* sp., *Nigrospora*sp., *Penicillium* sp.	*Taxus globosa*	[279]
			*Paraconiothyrium* sp.	*Taxus media*	[280]
			*Didymostilbe* sp.	*Taxus chinensis* var. *mairei*	[281]
			*Stemphylium sedicola*	*Taxus baccata*	[282]
			*Colletotrichum gloeosporioides*	*Tectona grandis*	[283]
			*Perenniporia tephropora*	*Taxus chinensis* var. *mairei*	[284]
			*Colletotrichum gloeosporioides*, *Fusarium proliferatum*, *Guignardia mangiferae*	*Taxus media*	[285]
			*Phoma betae*	*Ginkgo biloba*	[286]
			*Alternaria* sp.	*Corylus avellana*	[287]
			*Colletotrichum gloeosporioides*	*Moringa oleifera*	[288]
			*Penicillium* sp.	*Taxus chinensis*	[289]
			*Penicillium aurantiogriseum*	*Corylus avellana*	[290]
Toosendanin	Anticancer, antifeedant	*Melia azedarach*	Anonymous	*Melia azedarach*	[291,292]
Withanolide	Anticancer, cardiovascular disease, Alzheimer’s disease treatment	*Withania*sp.	*Taleromyces pinophilus*	*Withania somnifera*	[293]
Xanthatin	Antitumor	*Xanthium* spp.	*Paecilomyces* sp.	*Panax ginseng*	[294,295]

**Table 7:** Plant-derived quinones and xanthones produced by endophytic fungi.

Plant-Derived Quinones and Xanthones	Activities/Applications	Plant Source	Endophytic Source	Host Plant	References
1,7-dihydroxyxanthone	Antioxidant	*Weddellina squamulosa*	*Penicillium* sp.	*Avicennia*	[103]
Anthraquinone	Anticancer, antioxidant, laxative	*Digitalis viridiflora*, *Rumex* spp.	*Coniothyrium*sp.	*Salsola oppostifolia*	[316]
			*Aspergillus fumigatus*	*Rumex nepalensis*, *Rumex hastatus*	[317]
Emodin	Antibacterial, anti-inflammatory, antitumor, immunosuppressive	*Hypericum perforatum*, *Polygonum cuspidatum, Rheum* spp.	*Penicillium janthinellum*	*Melia azedarach*	[318,319]
			*Talaromyces* sp.	*Kandelia candel*	[320]
			*Aspergillus versicolor*	*Halimeda opuntia*	[321]
			*Fusarium solani*	*Rheum palmatum*	[322]
			*Eurotium chevalieri*	Mangrove	[323]
			*Alternaria alternata*	*Hypericum perforatum*	[324]
Eugenitin	Glucoamylase activation	*Syzygium aromaticum*	*Dothideomycetes* sp.	*Leea rubra*	[325]
Hypericin	Anti-depressant, antimicrobial, antiretroviral	*Hypericum perforatum*	*Chaetomium globosum*	*Hypericum perforatum*	[326]
			*Thielavia subthermophila*	*Hypericum perforatum*	[327]
			*Epicoccum nigrum*	*Hypericum perforatum*	[324]
Lapachol	Anticancer, antimicrobial, antiviral, anti-inflammatory, antiparasitic	*Tabebuia avellanedae*	*Alternaria* sp., *Alternaria alternata, Penicillium* sp.	*Tabebuia argentea*	[202]
			*Aspergillus niger*	*Tabebuia argentea*	[328]
Lawsone	Cytotoxic	*Lawsonia inermis*	*Gibberella moniliformis*	*Lawsonia inermis*	[329]
Pachybasin, Phomarin	Antibacterial, antiviral, bioagricultural agent	*Digitalis* spp., *Isoplexis isabelliana*	*Phoma sorghina*	*Tithonia diversifolia*	[330]
			*Coniothyrium* sp.	*Salsola oppostifolia*	[316]
Physcion (Parietin)	Antibiotics, antifungals, cytotoxic	*Hopea hainanensis*, *Rheum officinale*	*Aspergillus fumigatus*	*Cynodon dactylon*	[331]
			*Pleospora* sp.	*Imperata cylindrical*	[332]
			*Penicillium* sp.	*Hopea hainanensis*	[333]
			*Aspergillus terreus*	*Opuntia ficus-indica*	[334]
			*Cercosporella* sp.	*Schisandra chinensis*	[335]
			*Eurotiumchevalieri*	Mangrove	[323]
Pinselin (Cassiollin)	Cytotoxic	*Cassia occidentalis*	*Aspergillus sydowii*	*Scapania ciliata*	[336,337]
			*Phomopsis* sp.	*Paris polyphylla* var. *yunnanensis*	[338]
			*Phomopsis amygdali*	*Paris axialis*	[339]
			*Penicillium* sp.	*Sonneratia apetala*	[340]
Plumbagin	Anticancer	*Plumbago zeylanica*	*Cladosporium delicatulum*	*Terminalia pallida*	[341]
Questin	Antioxidant, allelopathic, herbicide	*Leea rubra*	*Dothideomycete*	*Leea rubra*	[325]
			*Eurotium rubrum*	*Hibiscus tiliaceus*	[342]
			*Eurotium cristatum*	*Sargassum thunbergii*	[343]
			*Aspergillus* sp.	*Pleioblastus amarus*	[344]
			*Phoma* sp.	*Phragmites communis*	[345]
			*Eurotium chevalieri*	Mangrove	[323]
Questinol	Anti-inflammatory, antibacterial	*Cassia* spp., *Polygonum* spp.	*Eurotium rubrum*	*Hibiscus tiliaceus*	[342]
			*Penicillium glabrum*	*Punica granatum*	[346]
			*Eurotium chevalieri*	Mangrove	[323]
Quinizarin	Cytotoxicity, antibacterial	*Rubia tinctorum*	*Epicoccum nigrum*	*Entada abyssinica*	[347]
Rhein	Anticancer, anti-inflammatory, antimicrobial, antioxidant, hepatoprotective, nephroprotective	*Rheum palmatum*	*Fusarium solani*	*Rheum palmatum*	[322]
Shikonin	Anti-inflammatory, anti-HIV, antimicrobial,	*Lithospermum erythrorhizon*	Anonymous	*Mammillaria hahniana*	[348]
			*Fusarium tricinctum*	*Lithospermum officinale*	[349]
Sterequinone C	Anti-inflammatory	*Stereospermum* spp.	*Penicillium* sp.	*Avicennia*	[103]
Tanshinone	Antibacterial, antifungal, anti-inflammatory, antihypertensive, antitumor	*Salvia* spp.	*Paecilomyces* sp.	*Panax ginseng*	[295]
			*Emericella foeniculicola*	*Salvia* spp.	[350]
			*Trichoderma atroviride*	*Salvia miltiorrhiza*	[351]
			*Phoma glomerata*	*Salvia miltiorrhiza*	[352]
			*Alternaria* sp.	*Salvia miltiorrhiza*	[353]
Torreyanic acid	Anticancer, cytotoxic	*Torreya taxifolia*	*Pestalotiopsis microspora*	*Torreya taxifolia*	[354]

**Table 8:** Miscellaneous plant-derived compounds produced by endophytic fungi.

Plant-Derived Compounds	Activities/Applications	Plant Source	Endophytic Source	Host Plant	References
3-Nitropropionic acid (beta-Nitropropionic acid)	Antimycobacterial, nematicidal, succinate dehydrogenase inhibitor	*Astragalus falcatus*, *Coronilla viminalis*, *Hippocrepis* sp., *Lotus, Scorpiurus* sp., *Securigera* sp.	*Melanconium betulinum*	Birches	[369,370]
			*Phomopsis phaseoli* (syn. *Diaporthe phaseolorum*)	Rainforest tree	[369]
			*Phomopsis* spp.	*Costus* sp.	[370]
			*Phomopsis longicolla*	*Trichilia elegans*	[371]
Asarone (Phenyl propane)	Antimicrobial	*Cinnamomum camphora*	*Muscodor tigerii*	*Cinnamomum camphora*	[372]
Azelaic acid (Saturated dicarboxylic acid)	Antimicrobial, anti-inflammatory, anticancer	Wheat, rye, barley	*Plectosphaerella cucumerina*	*Cynanchum auriculatum*	[373]
			*Aspergillus unguis*	*Enteromorpha* sp.	[374]
Cajaninstilbene acid	Antioxidant, anti-inflammatory, hypoglycemic, neuroprotective	*Cajanus cajan*	*Cytonaema* sp.	*Quercus* sp.	[375]
			*Alternaria*, *Fusarium oxysporum, Fusarium solani, Fusarium proliferatum*, *Neonectria macrodidym*	*Cajanus cajan*	[376]
Chlorogenic acid (5-O-caffeoylquinic acid) (Cinnamate conjugates)	Antimicrobial, antioxidant, antitumor, immunomodulatory, antiviral	*Arnica* spp., *Arctium lappa*, *Coffea canephora*, *Chrysanthemum coronarium*, *Schefflera heptaphylla*	Anonymous	*Artemisia indica*	[216]
			*Sordariomycete* sp.	*Eucommia ulmoides*	[377]
Eugenol (Phenyl propane)	Antimicrobial	*Syzygium aromaticum*	*Alternaria* sp.	*Rosa damascaena*	[378]
			*Annulohypoxylon stygium*	Anonymous	[379]
			*Rhizopus oryzae*	*Holarrhena pubescens*	[380]
			*Diaporthe* sp., *Neopestalotiopsis* sp.	*Cinnamomum loureiroi*	[381]
Digoxin	Cardiac, anticancer	*Digitalis lanata*	Anonymous	*Digitalis lanata*	[382]
Forskolin	Antiglaucoma, anti-HIV, antitumor	*Coleus forskohlii*	*Rhizoctonia bataticola*	*Coleus forskohlii*	[383]
Harpagide (Iridoid glycosides)	Anticancer, anti-inflammatory, Leishmanicidal	*Harpagophytum procumbens*	*Alternaria alternata*	*Scrophularia ningpoensis*	[384,385]
Myrtucommulones (Lactone)	Anticancer, anti-inflammatory	*Myrtus communis*	*Neofusicoccum australe* (teleomorph *Botryosphaeria australis*)	*Myrtus communis*	[386]
Naphthalene (Aromatic hydrocarbon)	Antibacterial, insect repellent	*Ancistrocladus tectorius*	*Muscodor vitigenus*	*Paullinia paullinioides*	[387,388]
			*Nodulisporium* sp.	*Erica arborea*	[389]
			*Phoma herbarum*	*Aegle marmelos*	[390]
Panaxynol or Falcarinol or Carotatoxin (Polyacetylene)	Anticancer	*Panax ginseng*, *Falcaria vulgaris*, *Daucus carota, Hedera* spp.	*Paecilomyces* sp.	*Panax ginseng*	[295]
Resveratrol (Stilbene polyphenol)	Antioxidant, anticancer, epigenetic modulation	*Vitis* spp.	*Alternaria* sp.*, Aspergillus* sp., *Botryosphaeria* sp., *Cephalosporium* sp., *Geotrichum* sp., *Mucor* sp., *Penicillium* sp.	*Polygonum cuspidatum*, *Vitis quinquangularis, Vitis vinifera*	[391]
Salidroside, p-tyrosol	Antioxidant, antihypoxic, adaptogenic	*Rhodiola rosea*	*Phialocephala fortinii*	*Rhodiola* sp.	[392]
Salvianolic acid (Polyphenol)	Antioxidant, cardiovascular, cerebrovascular diseases	*Salvia miltiorrhiza*	*Phoma glomerata*	*Salvia miltiorrhiza*	[352]
Tocopherol (Phenol)	Anti-influenza, antioxidant	*Ribes* sp.	*Aspergillus fumigatus*	*Cynodon dactylon*	[393]

## Data Availability

Data sharing is not applicable to this article.
